# Highly Osmotic Oxidized Sucrose-Crosslinked Polyethylenimine for Gene Delivery Systems

**DOI:** 10.3390/pharmaceutics13010087

**Published:** 2021-01-11

**Authors:** Jaehong Park, Kyusik Kim, Sohee Jeong, Migyeom Lee, Tae-il Kim

**Affiliations:** 1 Department of Biosystems & Biomaterials Science and Engineering, College of Agriculture and Life Sciences, Seoul National University, 1 Gwanak-ro, Gwanak-gu, Seoul 08826, Korea; pjh0520@snu.ac.kr (J.P.); kskim36@snu.ac.kr (K.K.); 2 Department of Agriculture, Forestry and Bioresources, College of Agriculture and Life Sciences, Seoul National University, 1 Gwanak-ro, Gwanak-gu, Seoul 08826, Korea; soheej97@snu.ac.kr (S.J.); wdfrost@snu.ac.kr (M.L.); 3 Research Institute of Agriculture and Life Sciences, Seoul National University, 1 Gwanak-ro, Gwanak-gu, Seoul 08826, Korea

**Keywords:** oxidized sucrose, polyethylenimine, crosslinking, gene delivery systems, osmolality, endocytosis, endosomal escape

## Abstract

In this work, highly osmotic oxidized sucrose-crosslinked polyethylenimine (SP2K) polymers were developed for gene delivery systems, and the transfection mechanism is examined. First, periodate-oxidized sucrose and polyethylenimine 2K (PEI2K) were crosslinked with various feed ratios via reductive amination. The synthesis was confirmed by ^1^H NMR and FTIR. The synthesized SP2K polymers could form positively charged (~40 mV zeta-potential) and nano-sized (150–200 nm) spherical polyplexes with plasmid DNA (pDNA). They showed lower cytotoxicity than PEI25K but concentration-dependent cytotoxicity. Among them, SP2K7 and SP2K10 showed higher transfection efficiency than PEI25K in both serum and serum-free conditions, revealing the good serum stability. It was found that SP2K polymers possessed high osmolality and endosome buffering capacity. The transfection experiments with cellular uptake inhibitors suggest that the transfection of SP2K polymers would progress by multiple pathways, including caveolae-mediated endocytosis. It was also thought that caveolae-mediated endocytosis of SP2K polyplexes would be facilitated through cyclooxygenase-2 (COX-2) expression induced by high osmotic pressure of SP2K polymers. Confocal microscopy results also supported that SP2K polyplexes would be internalized into cells via multiple pathways and escape endosomes efficiently via high osmolality and endosome buffering capacity. These results demonstrate the potential of SP2K polymers for gene delivery systems.

## 1. Introduction

Polyethylenimine (PEI) is one of most utilized cationic polymers for gene delivery systems [1]. PEI can condense genetic materials into nano-sized polyplexes via electrostatic interaction, which can be adsorbed on the cellular surface and efficiently internalized into cells [2,3]. It has also been reported that PEI can escape endosomebyendosome buffering ability after endocytosis due to the protonatable amine groups, leading to the efficient transfection [4,5]. However, highmolecularweight PEI can induce significant cytotoxicity, due to the non-degradability and high charge density, interacting with negatively charged cell membranes or biomolecules and causing disruption of the membranes, which forms aggregates and inhibits their functions [6,7]. One of strategies to overcome these drawbacks is the crosslinking of lowmolecularweight PEI with low cytotoxicity [8].

Biodegradable PEI derivatives with ester bonds via Michael addition-type crosslinking of low molecular weight PEIs with several diacrylates were developed for gene delivery systems, demonstrating low cytotoxicity and high transfection efficiency [9,10]. PEI-modified poly(β-amino ester) polymer was synthesized by crosslinking acrylate-terminated poly(β-amino ester)s with lowmolecularweight PEI, showing high transfection efficiency with little cytotoxicity [11].

Bioreducible PEI derivatives containing disulfide bonds were also prepared for gene delivery systems, which can be degraded in an intracellular environment containing a high concentration of glutathione (0.5–10.0 mM) [12]. Homobifunctional crosslinkers, dimethyl-3,3′-dithiopropionimidate (DTBP), and dithiobis(succinimidyl propionate) (DSP) were used for crosslinking of lowmolecularweight PEI [13]. The resultant polymers showed higher transfection efficiency and lower cytotoxicity than commercial transfection agents. Another bioreducible PEI was developed by crosslinking thiolated PEI synthesized through the reaction of methylthiirane with lowmolecularweight PEI. The resulting polymers demonstrated high transfection efficiency with low cytotoxicity [14].

Sucrose is a non-reducing disaccharide composed of glucose and fructose. Sucrose can be oxidized by periodate, generating multiple aldehyde groups that can react with amine groups via imine bond formation [15]. Therefore, oxidized sucrose has been utilized as a crosslinking agent for various applications, such as film and nanofibers [16,17].

Moreover, sucrose can induce high osmotic pressure [18]. It has been reported that osmotic stress to cells could stimulate caveolin-1 (Cav-1) phosphorylation via c-Src kinase activity [19], inducing caveolae-mediated endocytosis [20]. Based on this, several hyperosmotic polymeric gene delivery systems with sugar moieties, such as sorbitol [21], mannitol [22], or xylitol [23], have been developed, showing high transfection efficiency. 

Therefore, here we tried to synthesize crosslinked PEIs using oxidized sucrose as a crosslinker for gene delivery systems, and it was expected that their high osmotic sucrose moieties and unique structures would increase transfection efficiency through facilitated cellular uptake and endosomal escape. To the best of our knowledge, oxidized sucrose has never been used as a crosslinker for the synthesis of polymeric gene delivery systems. In this work, sucrose–polyethylenimine 2K (PEI2K) (SP2K) polymers were synthesized for gene delivery systems via the crosslinking of oxidized sucrose with lowmolecularweight PEI. The physicochemical properties of SP2K polymers and polyplexes were characterized. Their cytotoxicity and transfection efficiency were also evaluated, and the transfection mechanism was suggested for the first time.

## 2. Materials and Methods

### 2.1. Materials

Sucrose, polyethylenimine (PEI; molecular weight 2 kDa and 25 kDa), agarose, ethylenediaminetetraacetic acid (EDTA), ethidium bromide, genistein, nocodazole, 4-[5-(4-Chlorophenyl)-3-(trifluoromethyl)-1H-pyrazol-1-yl]-benzenesulfonamide (SC-236), and 3-[4,5-dimethylthiazol-2-yl]-2,5-diphenyltetrazolium bromide (MTT) were purchased from Sigma-Aldrich (St. Louis, MO, United States). Sodium periodate and sodium tetrahydroborate were purchased from Junsei (Tokyo, Japan). HPLC-grade water was purchased from Duksan (Ansan, Korea). Chlorpromazine was purchased from TCI (Tokyo, Japan). Reporter lysis buffer and luciferase assay system were purchased from Promega (Madison, WI, USA). Dulbecco’s modified Eagle’s medium (DMEM) + GlutaMax-I, Dulbecco’s phosphate buffered saline (DPBS), fetal bovine serum (FBS), 0.25% Trypsin–EDTA, penicillin/streptomycin (P/S), Quant-iT PicoGreen kit, YOYO-1 iodide, and LysoTracker Red DND-99 were purchased from Invitrogen-Gibco (Carlsbad, CA, USA). The BCA protein assay kit was purchased from PIERCE (Rockford, IL, USA). All other chemicals were purchased and used without any further purification.

### 2.2. Synthesis of Sucrose–PEI2k (SP2K)

SP2K polymers were synthesized by a crosslinking reaction between oxidized sucrose and PEI2K via reductive amination. First, sucrose (0.342 g, 1 mmol) and sodium periodate (0.642 g, 3 mmol) were dissolved in water, respectively. Then, sucrose solution and sodium periodate solution were mixed in the reaction bottles with continuous stirring. After 24 h of reaction in the dark at room temperature, the remaining sodium periodate was precipitated with an excess of acetone [24]. Supernatant was filtered and evaporated in a vacuum chamber for a day. Oxidized sucrose was obtained as a white solid after the following lyophilization. Then, PEI2K and oxidized sucrose were dissolved in water. The PEI2K solution was added dropwise to the oxidized sucrose solution for the crosslinking between them, with diverse feed ratios (aldehyde unit of oxidized sucrose/amine unit of PEI2K = 1:3, 1:5, 1:7, 1:10, and 1:15). The reaction was conducted for 24 h in the dark at room temperature. Sodium tetrahydroborate solution was mixed with the solution to reduce the formed imine bonds and unreacted aldehyde moieties. After 24 h of reaction, the reaction mixture was dialyzed with a dialysis membrane (MWCO = 3500 Da, Spectrum Laboratories, Inc., Rancho Dominguez, CA, United States) for 2 days against deionized water. The products, sucrose–PEI2Ks (SP2Ks) were obtained after lyophilization and named as SP2K3, SP2K5, SP2K7, SP2K10, and SP2K15, according to the feed ratios.

### 2.3. Characterization of SP2K

The synthesis of SP2K polymers was confirmed by ^1^H NMR (400 MHz JNM-LA400, JEOL, Tokyo, Japan) with a D_2_O solvent. 

Fourier-transform infrared spectrometry (FTIR) spectra of SP2K polymers were also obtained by an ATR (attenuated total reflectance) FTIR spectrometer (Nicolet 6700, Thermo Scientific, Waltham, MA, USA) in the range of 650–4000 cm^−1^, with 8 cm^−1^ intervals.

The molecular weights of SP2K polymers were determined by gel permeation chromatography (GPC) (YL-9100, Young Lin Instrument, Anyang, Korea) with Ultrahydrogel 250 column (Waters, Milford, MA, USA), with 1% formic acid as an eluent. Polyethylene glycols with various molecular weights were used as standards. The concentration of the samples and the flow rate were set to 10 mg/mL and 0.6 mL/min, respectively. 

Acid−base titration of SP2K polymers was performed to examine their endosome buffering capacities. Each SP2K polymer (10 mg) was dissolved in 10 mL of 0.1 M NaCl solution. The pH of the polymer solution was adjusted to 11.0 using 1 M NaOH and titrated to pH 3.0 with 0.1 M HCl. The pH changes of the solutions were measured by a pH meter (SevenEasy pH meter S20, Mettler-Toledo, Columbus, OH, USA). 

The osmolality of the SP2Ks was measured by using an osmometer (Micro sample osmometer A3320, Advanced Instruments, Norwood, MA, USA). Each polymer was dissolved in water at various concentrations (10–100 mg/mL). Then 20 μL of the sample was injected using a thermistor probe and the osmolarity was measured. The experiment was performed in triplicate.

### 2.4. Plasmid DNA Purification

Plasmid DNAs (pDNAs), pCN-Luci encoding luciferase and gWIZ-GFP (Genlantis, San Diego, CA, USA) encoding green fluorescent protein (GFP) were amplified with *Escherichia coli* DH5α and purified with Nucleobond Xtra Midi kit (Macherey-Nagel, Düren, Germany).

### 2.5. Agarose Gel Electrophoresis

The DNA condensation ability of the SP2K polymer was assessed by agarose gel electrophoresis. Agarose gel (0.7% *w*/*v*) was prepared in Tris–acetate–EDTA (TAE) buffer with ethidium bromide (0.5 μg/mL). The polyplexes (0.5 μg pDNA, 10 μL) were prepared in HEPES buffer (pH 7.4) at various weight ratios (polymer/pDNA) ranging from 0.1 to 0.5 by gently mixing pDNA solution in the polymer solutions. After 30 min of incubation at room temperature, the electrophoresis was run for 12 min (Mupid-2plus, Takara Bio, Kusatsu, Japan) after the loading of samples. The pDNA bands were observed by UV illuminator (GelDocXR+ gel documentation system, Bio-RAD, Hercules, CA, USA).

### 2.6. PicoGreen Assay

A PicoGreen assay was carried out using a Quant-iT PicoGreen kit to investigate the DNA condensation ability of SP2K quantitatively. The polyplexes with 0.5 μg pDNA were prepared in Tris-EDTA (TE) buffer (pH 7.4) at various weight ratios ranging from 0.1 to 2.0. After 30 min of incubation, the PicoGreen reagent was added to each polyplex solution. After a further 4 min of incubation, fluorescence (Ex = 480 nm, Em = 520 nm) was measured using a microplate reader (Synergy H1, BioTek, Winooski, VT, USA). The results were presented as relative values (% fluorescence of sample/fluorescence of pDNA) after triplicated measurements.

### 2.7. Average Sizes and Zeta-Potential Value Measurement

The average sizes and zeta-potential values of SP2K polyplexes were measured by using Zeta-sizer Nano ZS90 (Malvern Instruments, Malvern, UK) with an He–Ne ion laser (633 nm). SP2K polyplex solutions with 4 μg pDNA were prepared at various weight ratios ranging from 0.1 to 20.0 in deionized water. After 30 min of incubation, the average sizes and zeta-potential values were measured and the average values were presented after triplicated measurements.

### 2.8. Transmission Electron Microscopy (TEM) Observation

The morphology of SP2K polyplexes was observed by an energy-filtering transmission electron microscope (EF-TEM; LIBRA 120, Carl Zeiss, Oberkochen, Germany). SP2K polyplex solutions with 4 μg pDNA (weight ratio = 10) were prepared, and they were adsorbed on transmission electron microscopy (TEM) carbon grid plates for 1 min. The samples were then stained with uranyl acetate solution for 10 s. After removal and drying of residual solutions, the polyplex images were visualized by TEM with an accelerating voltage of 120 kV.

### 2.9. Cell Culture

HeLa (human cervical adenocarcinoma) cells were maintained in DMEM + GlutaMax-I medium, which was supplemented with 10% FBS and 1% penicillin/streptomycin in a humidified atmosphere containing 5% CO_2_ at 37 °C.

### 2.10. Cytotoxicity

The cytotoxicity of SP2K was measured using an MTT assay. The HeLa cells were seeded in 96-well plates at a density of 1 × 10^4^ cells/well. When the cells achieved 70–80% confluency, the cells were exposed to 100 μL of polymer solution with various concentrations (10–100 μg/mL) in serum-free medium for 4 h. After an exchange of medium with fresh medium containing 10% FBS, the cells were further incubated for 24 h. Then, 25 μL of MTT solution (2 mg/mL in DPBS) were treated to the cells for 2 h. After removal of each medium, DMSO (150 μL) was added to each well to dissolve the formazan formed by proliferating cells. The absorbance at 570 nm was measured by using a microplate reader. Results were presented as relative cell viabilities (RCV, %, absorbance of sample treated cells/absorbance of untreated control cells).

### 2.11. Transfection Experiments In Vitro

The transfection efficiency of SP2K was evaluated by using luciferase transgene expression assay. The HeLa cells were seeded in 24-well plates at a density of 5 × 10^4^ cells/well. At 70–80% of confluency, the medium of each well was exchanged with serum-free DMEM or DMEM containing 10% FBS. The cells were exposed to 100 μL of polyplex solutions with various weight ratios for 4 h. After an exchange of the medium with fresh medium (10% FBS), the cells were incubated for 48 h. Then the medium of each well was removed, and cells were rinsed twice with DPBS. Then, the cells were lysed with 120 μL of lysis buffer. After 30 min of incubation, the cell lysates were centrifuged (14,000 rpm, 10 min) at 4 °C. Luciferase activities of supernatants were measured by using luciferase assay system on a microplate reader. The total amounts of cellular proteins were measured with a BCA protein assay reagent kit for normalization. The results were presented in terms of relative light unit (RLU)/mg cellular protein. The experiment was performed in triplicate.

### 2.12. Green Fluorescence Protein (GFP) Expression 

The transfection efficiency of SP2K polymers was further examined by observing GFP transgene expression. HeLa cells were seeded in six-well plates at a density of 2 × 10^5^ cells/well and were grown to reach 70–80% confluency for 24 h. Before transfection, the media were exchanged with fresh, serum-free media. Then, the cells were treated with polyplex solutions (2 μg gWIZ-GFP; PEI25K weight ratio = 1; SP2K polymer weight ratios = 10 and 20). After 4 h of incubation, the transfection media were exchanged with fresh media (10% FBS). The cells were further incubated for 2 days. After washing cells with DPBS twice, the expression of GFP was observed by fluorescence microscopy (iRis Digital Cell Imaging System, Logos Biosystems, Anyang, Korea). The images were processed by the Image J program.

### 2.13. Cellular Uptake Mechanism Analysis

HeLa cells were seeded in 24-well culture plates at a density of 5 × 10^4^ cells/well. After achieving 70–80% of confluency for 24 h of incubation, pre-treatment was performed for 30 min with serum-free media containing various cellular uptake inhibitors: chlorpromazine (clathrin-mediated endocytosis inhibitor, 10 μM), nocodazole (microtubule-mediated endocytosis inhibitor, 33 μM), genistein (caveolae-medicated endocytosis inhibitor, 200 μM), or SC-236 (COX-2 inhibitor, 5–30 μM). After washing with DPBS, polyplexes were treated for 4 h. After media exchange, the cells were allowed to grow for further 48 h. The following assays were conducted identically to the above transfection assay. The results were presented in terms of RLU/mg protein. The experiment was performed in triplicate.

### 2.14. Intracellular Trafficking of Polyplex

Intracellular trafficking of SP2K polyplexes was observed by using a confocal laser scanning microscope (CLSM). HeLa cells were seeded in confocal imaging dishes at a density of 3 × 10^5^ cells/dish. The media were exchanged with fresh serum-free DMEM after 24 h of incubation. Then, the cells were exposed to the polyplex solutions (3 μg pDNA labeled by YOYO-1 iodide; PEI25K weight ratio = 1, SP2K7 weight ratio = 10). The cells were washed with DPBS twice after 4 h of incubation and stained by LysoTracker Red DND-99 (500 nM). After fixation of cells with 4% paraformaldehyde, cell nuclei were stained by 4′,6-diamidine-2′-phenylindole dihydrochloride (DAPI) solution (14.3 μM). After washing with DPBS twice, cell images were observed by CLSM (SP8X STED, Leica, Wetzlar, Germany) and processed by the LAS X program.

## 3. Results and Discussion

### 3.1. Synthesis of Sucrose–PEI2K (SP2K)

Sucrose is a non-reducing disaccharide composed of glucose and fructose (α-D-glucopyranosyl-(1→2)-β-D-fructofuranoside). After periodate oxidation, diols of sucrose (–OH of C2 and C4 of glucose, –OH of C3 and C4 of fructose) can be converted to dialdehydes by the oxidative cleavage of C–C bonds with the evolution of formic acid [17]. Then, aldehydes of oxidized sucrose can be reacted with amines of PEI2K via the formation of imine bonds. Therefore, oxidized sucrose could be used as a crosslinker for the synthesis of sucrose–PEI2Ks (SP2Ks). These crosslinking reactions were performed with diverse feed ratios (aldehyde unit of oxidized sucrose/amine unit of PEI2K = 1:3, 1:5, 1:7, 1:10, and 1:15), and the synthesized polymers were named SP2K3, SP2K5, SP2K7, SP2K10, and SP2K15, respectively, according to the feed ratios. The synthesis scheme was shown in Figure S1. 

**Figure 1:**
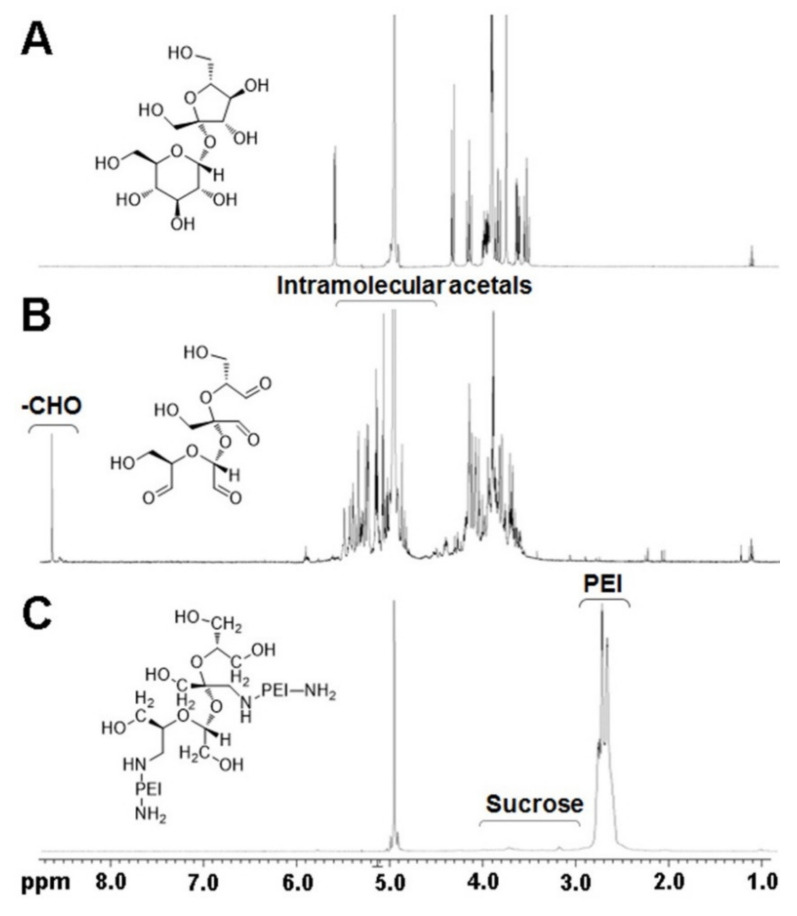
^1^H NMR spectra and chemical structures of (**A**) sucrose, (**B**) oxidized sucrose, and (**C**) sucrose- polyethylenimine (SP2K) polymer.

Figure 1A,B show the ^1^H NMR spectra of sucrose and oxidized sucrose, respectively. In Figure 1B, the proton peak of aldehyde (–CHO) was detected at δ 8.6, confirming that diols of sucrose were oxidized to aldehydes. Intramolecular acetal proton peaks appeared from 4.5–5.5 ppm. These acetal bonds were formed by the reaction of aldehyde groups of oxidized sucrose with remaining hydroxyl groups [25]. Other proton peaks of oxidized sucrose appeared at δ 3.5–4.5. After imine bond formation between aldehyde groups of oxidized sucrose and amine groups of PEI2K, unreacted aldehyde groups and the formed imine groups were reduced by NaBH_4_. In Figure 1C, the proton peaks of PEI2K (–CH_2_CH_2_NH–) and sucrose were observed at δ 2.5–2.9 and δ 3.1–4.0, respectively, confirming that crosslinking reaction between PEI2K and sucrose was successfully performed. According to the comparison of proton peak integrals, the composition ratios (PEI2K/sucrose) of SP2K polymers were calculated and are presented in Table 1. The composition ratios increased with the increase of the initial feed amount of PEI2K. It was also confirmed by the absence of aldehyde proton peak (δ 8.6) that all unreacted aldehyde groups were reduced.

The molecular weights of SP2K polymers were measured by GPC (Table 1). The molecular weights decreased with increasing amounts of PEI2K. SP2K3 showed the highest molecular weight (16.9 kDa), probably due to the lowest stoichiometric ratio of aldehyde of oxidized sucrose and amine of PEI2K in crosslinking reaction. The GPC chromatograms of SP2K polymers are presented in Figure S2. 

The synthesis of SP2K polymers was further examined by FTIR. In Figure S3, characteristic absorption bands (3000–4000 cm^−1^ = OH stretching, 2800–3000 cm^−1^ = CH stretching) were observed in sucrose, oxidized sucrose, and SP2K. Oxidized sucrose showed CO stretching (1700 cm^−1^), confirming the oxidation of sucrose by periodate. After crosslinking reactions between oxidized sucrose and PEI2K, new characteristic absorption peaks of PEI were additionally observed, and CO stretching peaks disappeared, meaning the reaction was performed successfully (~3300 cm^−1^ = NH stretching, ~1600 cm^−1^ = NH bending, 1460 cm^−1^ = CH bending, and 1300 cm^−1^ = CN stretching) [26,27].

### 3.2. Acid–Base Titration

Endosome buffering capacities of SP2K polymers were examined by acid–base titration. It is thought that the high endosome buffering capacity of polymeric gene delivery carriers can induce efficient endosomal escape of polyplexes, leading to the high transfection efficiency [28]. It is expected that SP2K polymers also would possess high endosome buffering capacities because of their PEI2K moieties, which contain secondary and tertiary amines. As shown in Figure S4, endosome buffering capacities of SP2K polymers were similar to that of PEI2K and higher than that of PEI25k. Therefore, this result demonstrated the potential of SP2K polymers for efficient gene delivery systems.

### 3.3. pDNA Condensation Ability of SP2K Polymers

**Figure 2:**
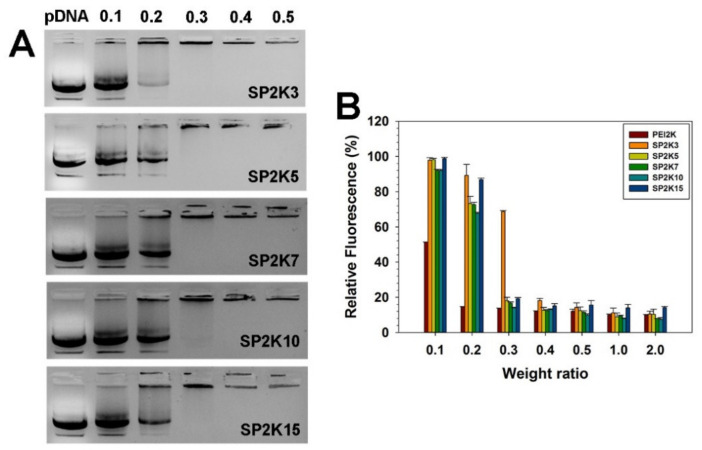
(**A**) Agarose gel electrophoresis result and (**B**) PicoGreen assay result of SP2K polyplexes. Numbers in (**A**) refer to the weight ratios of the polyplexes. Error bars in (**B**) indicate the standard deviations (*n* = 3).

Agarose electrophoresis was performed to investigate the pDNA condensation ability of SP2K polymers by electrostatic interaction. SP2K polyplexes were formed at various weight ratios ranging from 0.1 to 0.5. As shown in Figure 2A, migration of pDNA bands from all polyplexes was reduced with the increase of the polyplex weight ratios, and no migration of pDNA bands was observed from a weight ratio of 0.4, irrespective of crosslinking degrees. This result shows that all SP2K polymers could condense pDNA efficiently even at a low weight ratio, and that sucrose moiety did not hamper the pDNA condensation of SP2K polymers. 

The pDNA condensation ability of SP2K polymers was further examined by PicoGreen assay (Figure 2B). PicoGreen reagent is used to detect double stranded DNA (dsDNA), which can emit fluorescence quantitatively by binding to dsDNA [29]. The fluorescence values of all SP2K polyplexes decreased to less than 20% from a weight ratio of 0.3, except for SP2K3, which means that SP2K polymers could coat pDNA efficiently even at a low weight ratio during the condensation process. This result corresponds well to the previous gel electrophoresis results. It is noteworthy that SP2K7 and SP2K10 polyplexes showed lower fluorescence values than others, suggesting their stronger pDNA condensation abilities.

pDNA protection ability of SP2K polyplexes from serum nuclease was also examined. In Figure S5, pDNA was degraded in 50% FBS condition, showing the nuclease activity of serum nuclease. However, no significant pDNA fragment bands were observed in any SP2K polyplexes, like PEI25K polyplexes, after incubation with 50% FBS. This result shows the good pDNA protection ability of SP2K polymers via compact condensation.

In addition, polyplexes interact with various polyanionic molecules during transfection, which may induce the dissociation of polyplexes. Therefore, a heparin competition assay was further performed to investigate the pDNA binding ability of SP2K polymers. As shown in Figure S6, all SP2K polyplexes began to release pDNA at high heparin concentration (1.0–1.1 mg/mL) by competitive interaction of SP2K polymers with heparin. This result demonstrates the strong pDNA binding ability of SP2K polymers. However, pDNA protection and the release ability of polymeric gene delivery systems are mostly inversely related, and the maximum transfection efficiency can be obtained in an optimal balance between them [30].

### 3.4. Characterization of SP2K Polyplexes

**Figure 3:**
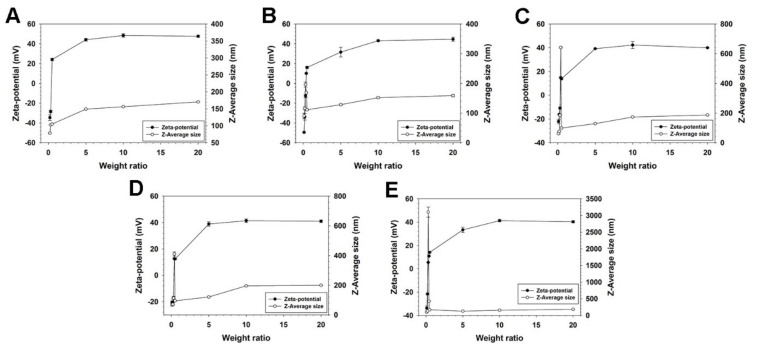
*Z*-average size and zeta-potential value measurement results of (**A**) SP2K3, (**B**) SP2K 5, (**C**) SP2K7, (**D**) SP2K10, and (**E**) SP2K15 polyplexes. Error bars indicate the standard deviations (*n* = 3).

After confirmation of the pDNA condensation ability of SP2K polymers, the *Z*-average sizes and zeta-potential values of SP2K polyplexes were measured by using a zeta-sizer. SP2K polyplexes showed negative zeta-potential values (−49.5~−22.1 mV) at a weight ratio of 0.1, and they rapidly turned to positive values (10.1–14.8 mV) at a weight ratio of 0.4 (Figure 3). Above a weight ratio of 5, all zeta-potential values of SP2K polyplexes became higher than 30 mV, due to the addition of cationic polymers. This result indicated that SP2K polymers could form positively charged polyplexes with pDNA, even from low weight ratios. In the case of size measurement, it was observed that all SP2k polyplexes showed abrupt size increases at the low weight ratio range (0.3–0.5), indicating the formation of large aggregates. This was probably due to the hydrophobic interaction between electrically neutralized polyplexes, inducing large aggregate formation according to their zeta-potential value changes. Then, the aggregates would be dissociated to smaller nanoparticles by electrostatic repulsion between positively charged polyplexes with the increase of polyplex weight ratios. At a weight ratio of 20, all SP2K polyplexes displayed 150–200 nm sizes and 40–45 mV zeta-potential values, meaning the formation of stable nano-sized polyplexes. These results suggested that SP2K polymers could form positively charged and nano-sized polyplexes with pDNA, which are suitable for adsorption onto the cell membrane and efficient cellular uptake [31]. Table S1 shows the PDI values of SP2K polyplex sizes. Although the values were somewhat high at low weight ratios, probably due to the instable polyplex formation, they were reduced to less than 0.2 at high weight ratios (>5), meaning that SP2K polymers could form homogeneous, nano-sized polyplexes with acceptable PDI values [32]. 

**Figure 4:**
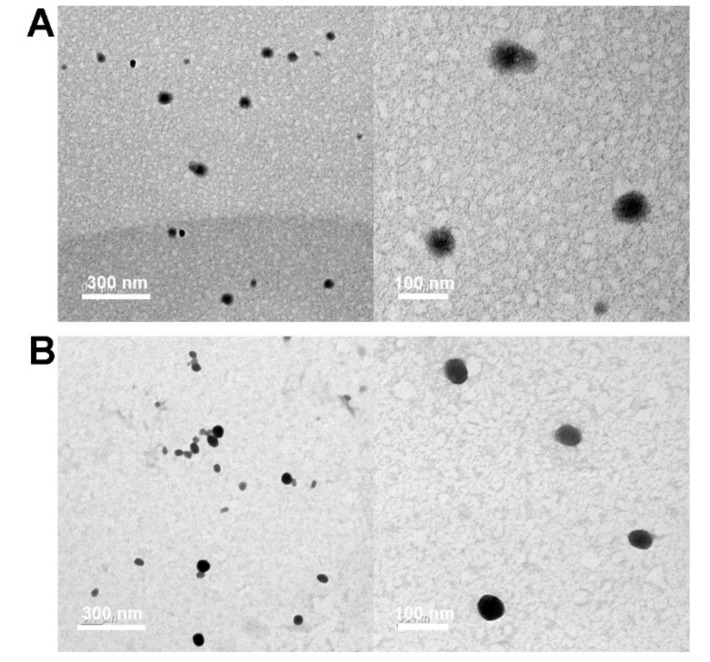
Transmission electron microscopy (TEM) images of (**A**) SP2K7 polyplexes and (**B**) SP2K10 polyplexes.

The morphology of SP2K polyplexes was also observed by TEM. SP2K7 and SP2K10 polyplexes were formed at a weight ratio of 10 and used for TEM observation. As shown in Figure 4, SP2K polyplexes displayed spherical nanoparticles with sizes ranging from 50 to 100 nm. It was thought that the dry TEM conditions would induce the shrinkage of polyplexes (smaller observed sizes) compared to the aqueous zeta-sizer condition.

### 3.5. Cytotoxicity

**Figure 5:**
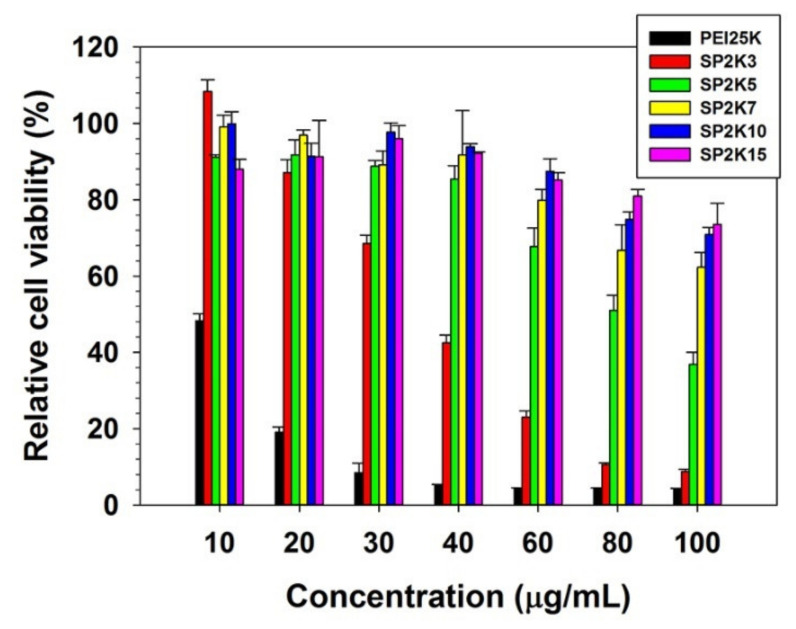
MTT assay result of SP2K polymers in HeLa cells. Error bars indicate the standard deviations (*n* = 3).

The cytotoxicity of SP2K polymers was examined by MTT assay. As shown in Figure 5, PEI25K-treated cells exhibited a significant decrease in cell viability (48.3% relative cell viability (RCV) at 10 μg/mL), indicating the high cytotoxicity of PEI25K in HeLa cells. SP2K-treated cells showed higher cell viability than PEI25K-treated cells, but also a concentration-dependent decrease in cell viability. In addition, the cytotoxicity of SP2K7 and SP2K10 was examined in A549 cells and RAW264.7 cells (Figure S7). They showed similar trends to the HeLa cell results. Interestingly, it was found that SP2Ks with a lower PEI2K portion could induce higher cytotoxicity. This is probably due to the higher molecular weights of SP2Ks with lower PEI2K portions [33].

### 3.6. Transfection Efficiency

**Figure 6:**
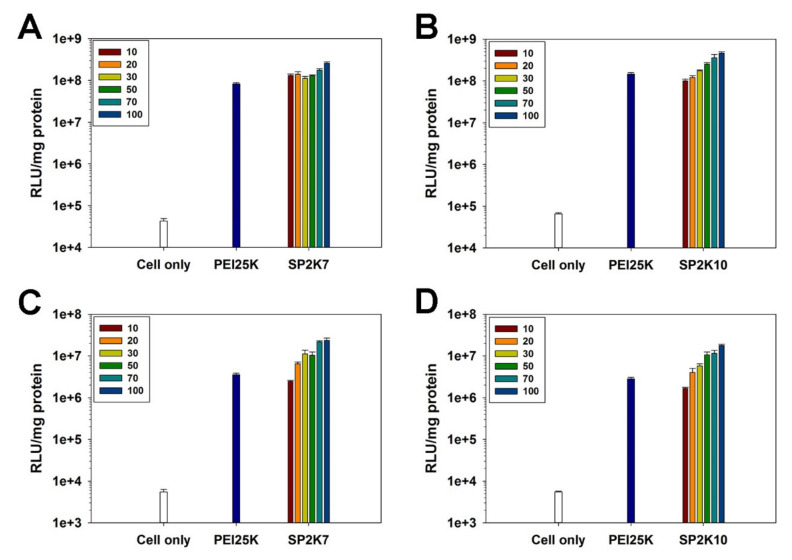
Transfection efficiency of (**A**,**C**) SP2K7 and (**B**,**D**) SP2K10 in HeLa cells. Transfection experiments were performed in (**A**,**B**) serum-free conditions and (**C**,**D**) serum conditions. Numbers in boxes indicate the weight ratios of the polyplexes. Error bars indicate the standard deviations (*n* = 3).

Luciferase transgene expression assay was performed to investigate the transfection efficiency of SP2K polymers in HeLa cells (Figure 6). According to the cytotoxicity results, less cytotoxic SP2K7 and SP2K10 were used for the experiments. Both polymers showed increased transfection efficiency with the increase of polyplex weight ratios, and usually displayed higher transfection efficiency than PEI25K above a weight ratio of 20 in serum-free conditions (Figure 6A,B). In serum conditions (Figure 6C,D), although the transfection efficiencies of all polymers decreased due to interaction with the serum proteins, SP2K7 and SP2K10 still could show the higher transfection efficiencies than PEI25K, suggesting their high serum stability. These results indicate that the sucrose moiety and special crosslinked structure of SP2K polymers may contribute to their high transfection efficiency.

**Figure 7:**
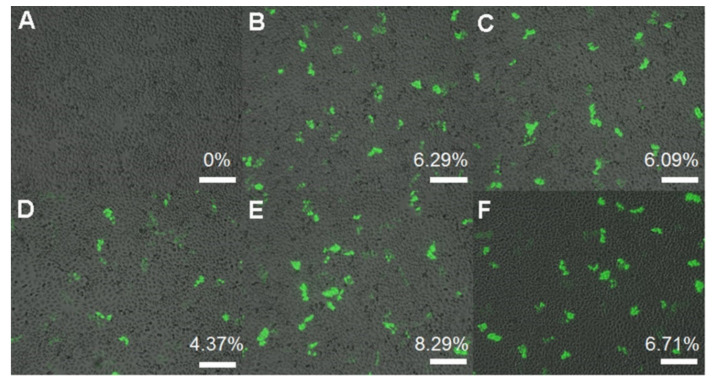
GFP transfection observation by fluorescence microscopy in HeLa cells. (**A**) cell only, (**B**,**E**) SP2K7 polyplexes, (**C**,**F**) SP2K10 polyplexes, and (**D**) PEI25K polyplexes. Transfection experiments were performed at weight ratios of (**B**,**C**) 10 and (**E**,**F**) 20. GFP expression (%) was calculated by the Image J program.

GFP transgene expression was also observed by using fluorescence microscopy to further investigate the transfection efficiency of SP2K polymers in HeLa cells. As shown in Figure 7D, PEI25K polyplex-treated cells showed only 4.37% GFP expression, but SP2K7 and SP2K10 polyplex-treated cells displayed higher GFP expression (6.29–8.29% and 6.09–6.71%, respectively). This result well corresponded to the luciferase transgene expression assay.

To examine the transfection mechanism, osmolality measurements, transfection experiments using several inhibitors, and intracellular trafficking observations were performed. Considering their cytotoxicity, only polyplex weight ratios of 10 or 20 were used for further transfection experiments.

### 3.7. Osmolarity Measurement

It was reported that the high osmolality of polymeric gene delivery carriers could induce endosomal escape of the polyplexes and expression of cyclooxygenase-2 (COX-2) facilitating the caveolae-mediated endocytosis of the polyplexes [19,20]. Therefore, the osmolality of SP2K polymers was examined using an osmometer, because SP2K polymers also possessed high osmotic sugar moieties in the backbone. As shown in Figure S8, SP2K7 and SP2K10 displayed higher osmolality than PEI2K and PEI25K, as expected. This osmolality measurement result suggests that SP2K polymers would cause a hyperosmotic environment near the cell membrane or within the endosome, finally leading to the high transfection efficiency.

### 3.8. Transfection Mechanism 

**Figure 8:**
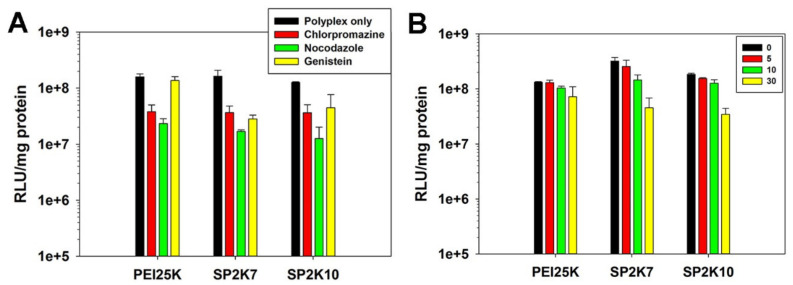
Transfection results using cellular uptake inhibitors in HeLa cells. The numbers in (**B**) indicate the concentration of SC-236 (μM). Error bars indicate the standard deviations (*n* = 3).

The transfection mechanism of SP2K polymers was investigated by using several cellular uptake inhibitors (genistein = caveolae-medicated endocytosis inhibitor, nocodazole = microtubule-mediated endocytosis inhibitor, and chlorpromazine = clathrin-mediated endocytosis inhibitor). As shown in Figure 8A, the transfection efficiency of PEI25K was significantly decreased in the presence of chlorpromazine (to 24%) and nocodazole (to 15%). In the case of SP2K polymers, the transfection efficiencies similarly decreased in the presence of chlorpromazine (SP2K7: to 22%, SP2K10: to 29%) and nocodazole (SP2K7: to 10%, SP2K10: to 10%). This result means that transfection of SP2K polyplexes would progress by clathrin- and microtubule-mediated endocytosis. It is known that nocodazole can inhibit not only microtubule-mediated endocytosis, but also the progression of early endosomes to late endosomes [34], which can decrease the transfection efficiency of endocytosed polyplexes. 

However, in genistein (caveolae-mediated endocytosis inhibitor) conditions, the transfection efficiencies of SP2K polymers (SP2K7: to 17%, SP2K10: to 35%) decreased much more than that of PEI25K (to 86%), suggesting that SP2K polyplexes would be more internalized into cells via caveolae-mediated endocytosis, probably due to the induction of caveolae formation by high osmotic pressure.

In addition, the transfection experiments were performed to confirm the effect of the high osmolality of SP2K to transfection by using SC-236 (COX-2 inhibitor) [35]. In Figure 8B, the transfection efficiency of PEI25K was gradually decreased with the increase of SC-236 concentration (to 54% at 30 μM). However, SP2K polymers showed significantly reduced transfection efficiencies (SP2K7: to 14%, SP2K10: to 18%). This result corresponds well to the above results, and means that highly osmotic SP2Ks can induce COX-2 expression, which can facilitate the caveolae-mediated endocytosis. 

Therefore, it is concluded that the transfection of SP2K polyplexes would progress by multiple endocytosis pathways, including caveolae-mediated endocytosis induced by high osmotic pressure.

### 3.9. Intracellular Trafficking of SP2K Polyplexes

**Figure 9:**
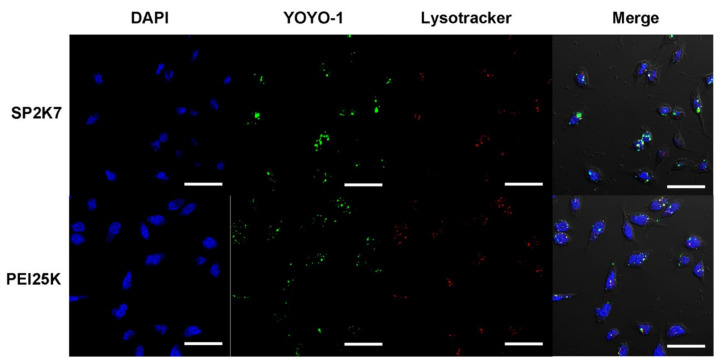
Confocal microscopy observation of SP2K7 polyplexes in HeLa cells (scale bar = 50 μm). PEI25K was used as a control.

Intracellular trafficking of SP2K7 polyplexes (YOYO-1 iodide) was examined by using confocal microscopy. Cell nuclei were stained by DAPI and acidic intracellular vesicles were stained by LysoTracker. As shown in Figure 9, both PEI25K and SP2K7 polyplexes were internalized into cells, and many of them were co-localized with acidic vesicles, meaning that they were internalized by endocytosis. However, more SP2K7 polyplexes were observed outside of acidic vesicles than PEI25K polyplexes. Based on this observation, it is elucidated that SP2K7 polyplexes with high endosome buffering capacity and osmolality would induce the efficient endosomal escape, and that SP2K7 polyplexes would be internalized into cells via neutral caveosomes [36], as mentioned above.

After endocytosis, it is thought that SP2K polyplexes would undergo spontaneous dissociation due to thermodynamics or via competitive displacement of pDNA by intracellular anionic molecules [37]. There is a possibility that pDNA release may be partially facilitated by glycosidase-mediated hydrolysis of SP2K polymer acetal linkages [38]. Considering the structural similarity of SP2K polymers with PEI, whether or not the polyplexes were dissociated, some portions would enter the nucleus passively during mitosis [39], like PEI derivatives. The significantly reduced transfection efficiency of SP2K polyplexes with nocodazole treatment also indicates that microtubules may play a role in nuclear trafficking of the polyplexes [40]. In addition, hydroxyl-rich sugar moieties of SP2K polyplexes may improve the nuclear trafficking of the polyplexes by interaction with carbohydrate receptors found on the nuclear envelope [41].

## 4. Conclusions

Sucrose–PEI2K (SP2K) polymers were synthesized for gene delivery systems by crosslinking of lowmolecularweight PEI2K and oxidized sucrose. SP2K polymers could form positivelycharged and nano-sized spherical polyplexes with pDNA at low weight ratios. The cytotoxicity of SP2K polymers was lower than that of PEI25K, but was concentration-dependent. SP2K polymers showed greater transfection efficiency than PEI25K in both serum-free and serum conditions. It was elucidated that the transfection of SP2K polymers would progress via multiple endocytosis pathways, including caveolae-mediated endocytosis induced by the high osmolality of SP2K polymers, and that the high osmolality and endosome buffering capacity would contribute to the high transfection efficiency of SP2K polymers by acid–base titration, osmolality measurement, and transfection experiment using inhibitors. Confocal microscopy observation showed that SP2K polyplexes would be efficiently internalized into cells and escape from endosome. These results reveal the transfection mechanism of SP2K polymers, and suggest their potential for efficient gene delivery systems.

## Figures and Tables

**Table 1:** Characterization of SP2K polymers. Molar ratios mean the ratio (amine of PEI2K/aldehyde of oxidized sucrose).

SP2K	Initial Feed Molar Ratio	Experimental Molar Ratio ^a^	Mw ^b^ (kDa)	PDI
SP2K3	3	0.37	16.9	1.71
SP2K5	5	0.53	15.5	1.71
SP2K7	7	0.65	11.1	2.01
SP2K10	10	0.83	10.4	1.86
SP2K15	15	1.02	8.7	1.83

^a^ Experimental molar ratios were determined by ^1^H NMR. ^b^ Mws (weight-average molecular weights) were measured by gel permeation chromatography (GPC).

## Data Availability

The data presented in this study are available in article or Supplementary Materials  here.

## Supplementary Materials

The following are available online at https://www.mdpi.com/1999-4923/13/1/87/s1, Figure S1: Synthesis scheme of SP2K polymers; Figure S2: GPC chromatograms of SP2K polymers; Figure S3: FTIR spectra of SP2K polymers; Figure S4: Acid–base titration of SP2K polymers; Figure S5: Protection ability of SP2K polyplexes from serum nuclease; Figure S6: Heparin competition assay of SP2K polyplexes; Figure S7: Cytotoxicity of SP2K7 and SP2K10 in (**A**) A549 cells and (**B**) RAW264.7 cells; Figure S8: Osmolality of SP2K polymers; Table S1: Average PDI values of SP2K polyplexes (*n* = 3).
