# Supplementary material for: Highly Osmotic Oxidized Sucrose-Crosslinked Polyethylenimine for Gene Delivery Systems
Source: Pharmaceutics. 2021 Jan 11;13(1):87. doi: 10.3390/pharmaceutics13010087 (PMC7826834; doi:10.3390/pharmaceutics13010087)
Supplement: Supplementary file 1 [file pharmaceutics-13-00087-s001.pdf]

pharmaceutics

Supplementary Materials: Highly Osmotic Oxidized Sucrose-Crosslinked Polyethylenimine for Gene Delivery Systems

Jaehong Park, Kyusik Kim, Sohee Jeong, Migyeom Lee and Tae-il Kim

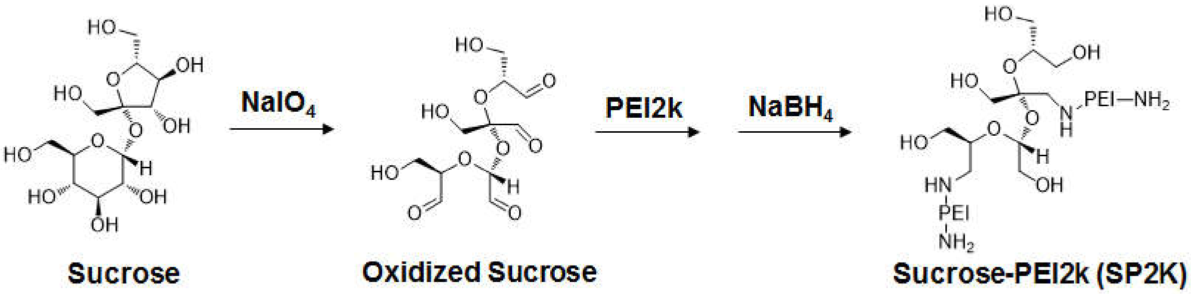

Figure S1. Synthesis scheme of SP2K polymers.

A) SP2K3

B) SP2K5

C) SP2K7

D) SP2K10

E) SP2K15

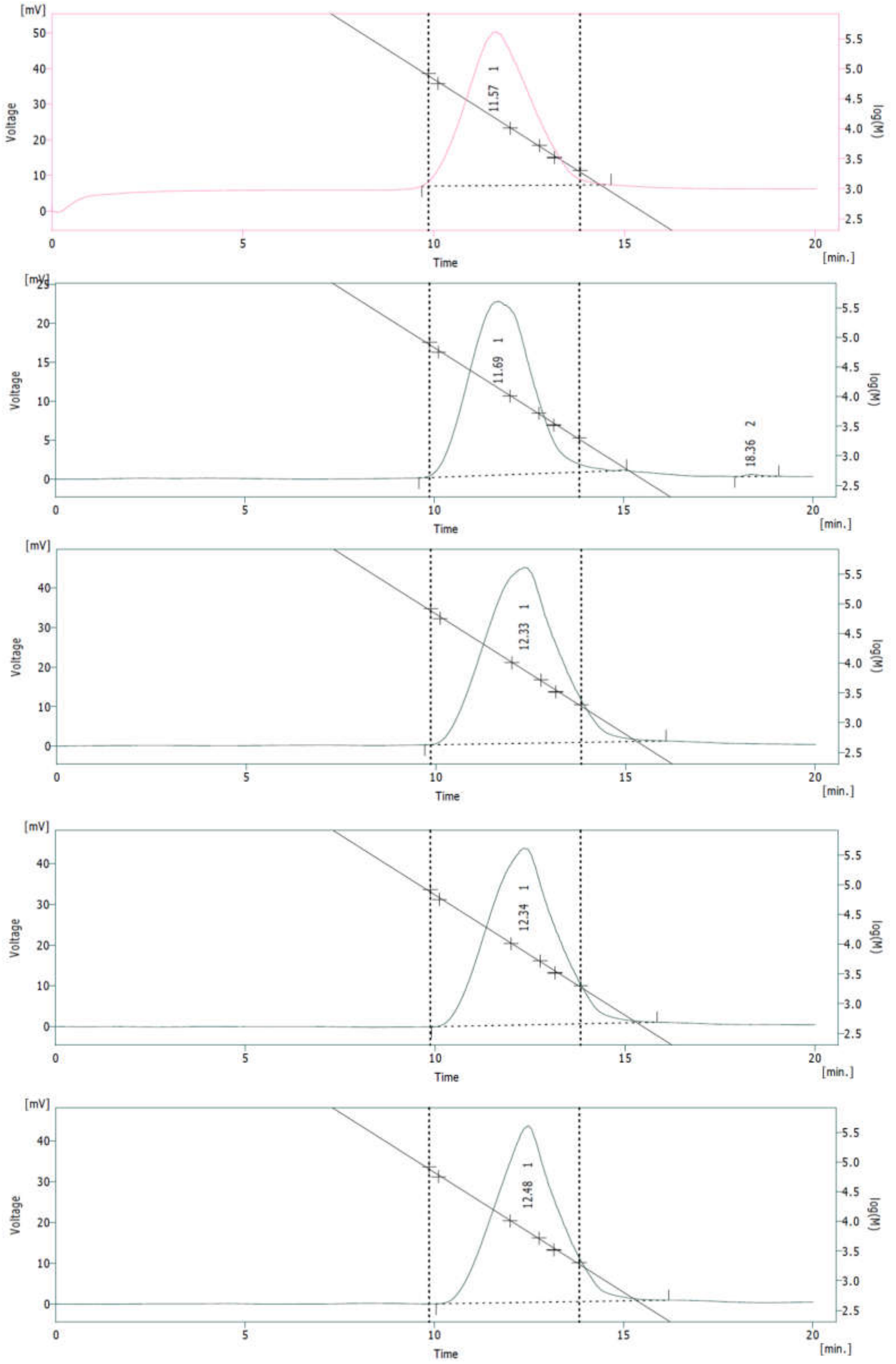

Figure S2. GPC chromatograms of SP2K polymers.

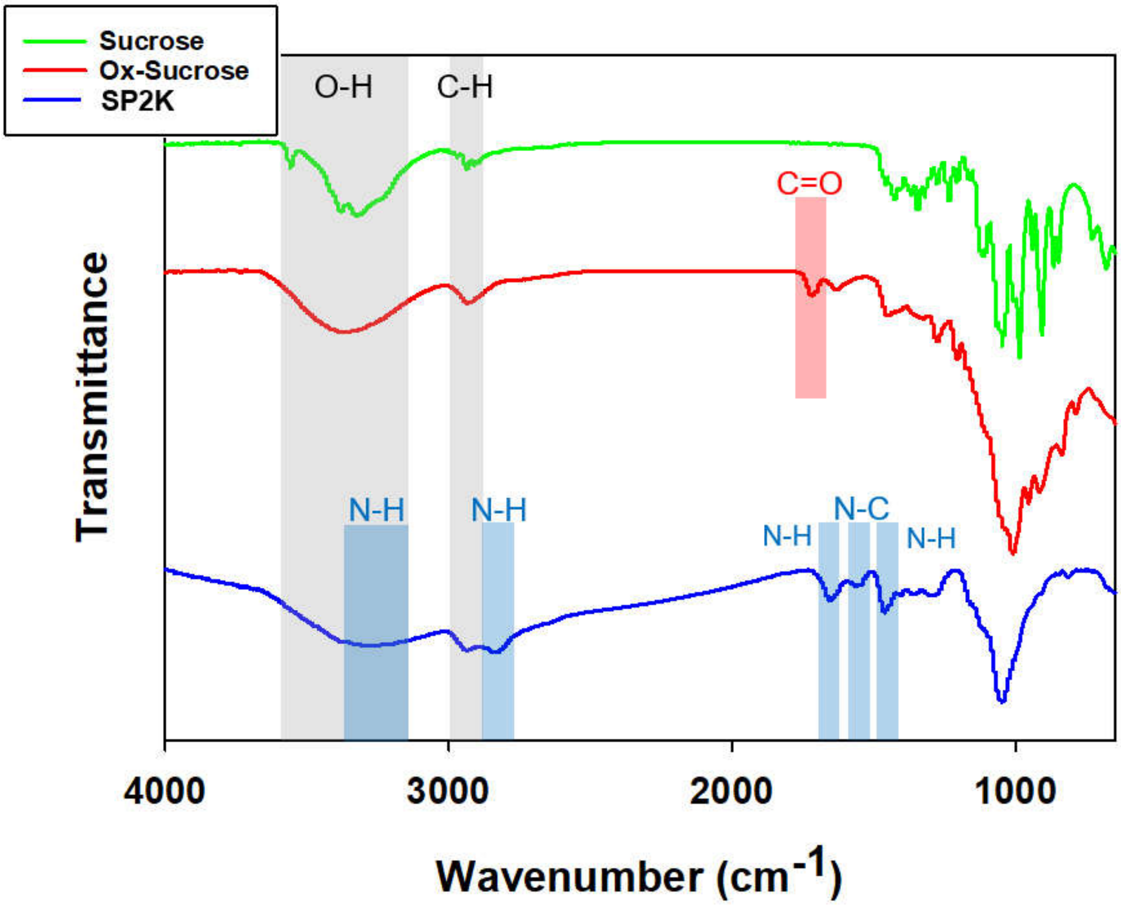

Figure S3. FT-IR spectra of SP2K.

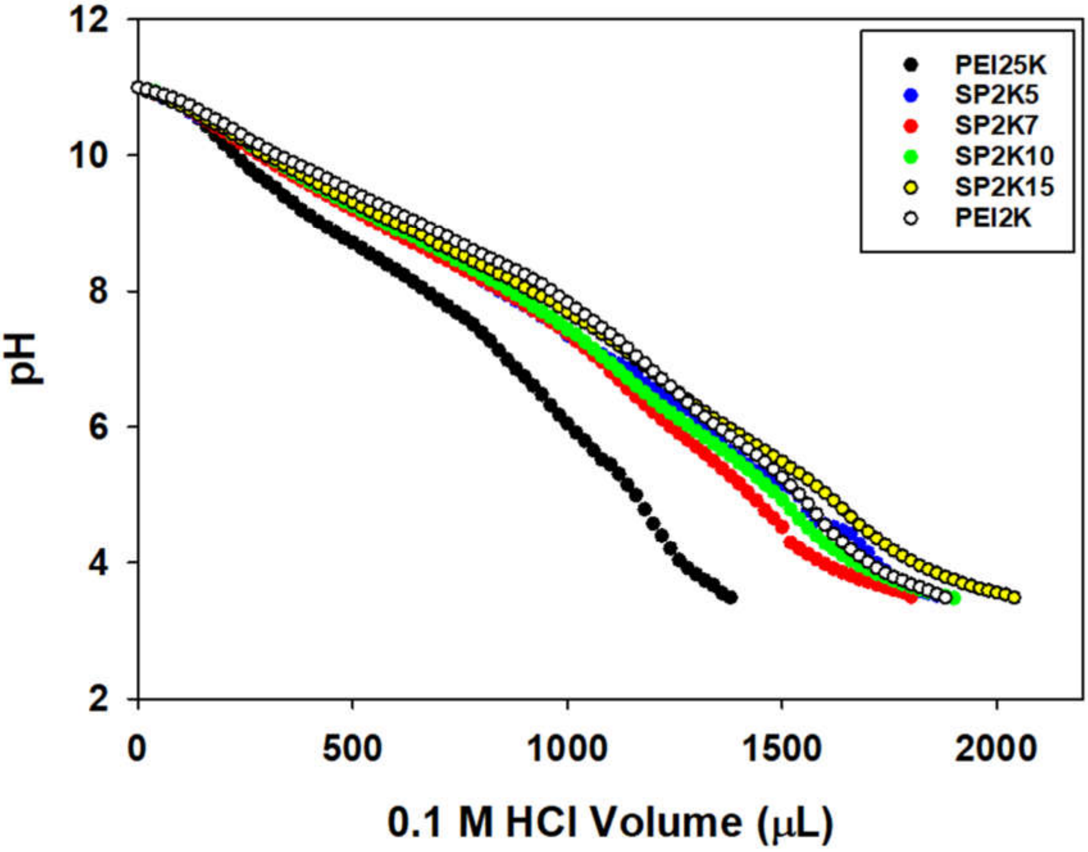

Figure S4. Acid-base titration of SP2K polymers

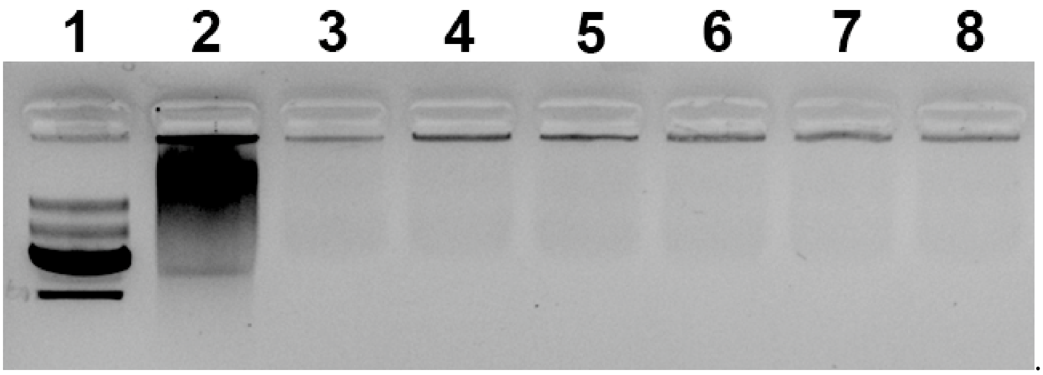

Figure S5. Protection ability of SP2K polyplexes from serum nuclease. 1) pDNA only, 2) pDNA + 50% FBS, 3) PEI25K polyplex + 50% FBS, 4) SP2K3 polyplex + 50% FBS, 5) SP2K5 polyplex + 50% FBS, 6) SP2K7 polyplex + 50% FBS, 7) SP2K10 polyplex + 50% FBS, and 8) SP2K15 polyplex + 50% FBS.

The SP2K polyplexes (0.5 μg pDNA) were prepared in HEPES buffer (pH 7.4) at a weight ratio of 10 after 30 min of incubation at room temperature. PEI25K polyplexes were prepared at a weight ratio of 1. pDNA was also used as a control. Each sample was incubated with 50% FBS at 37 °C. After 30 min of incubation, the agarose gel electrophoresis was run by the identical method to Section 2.5.

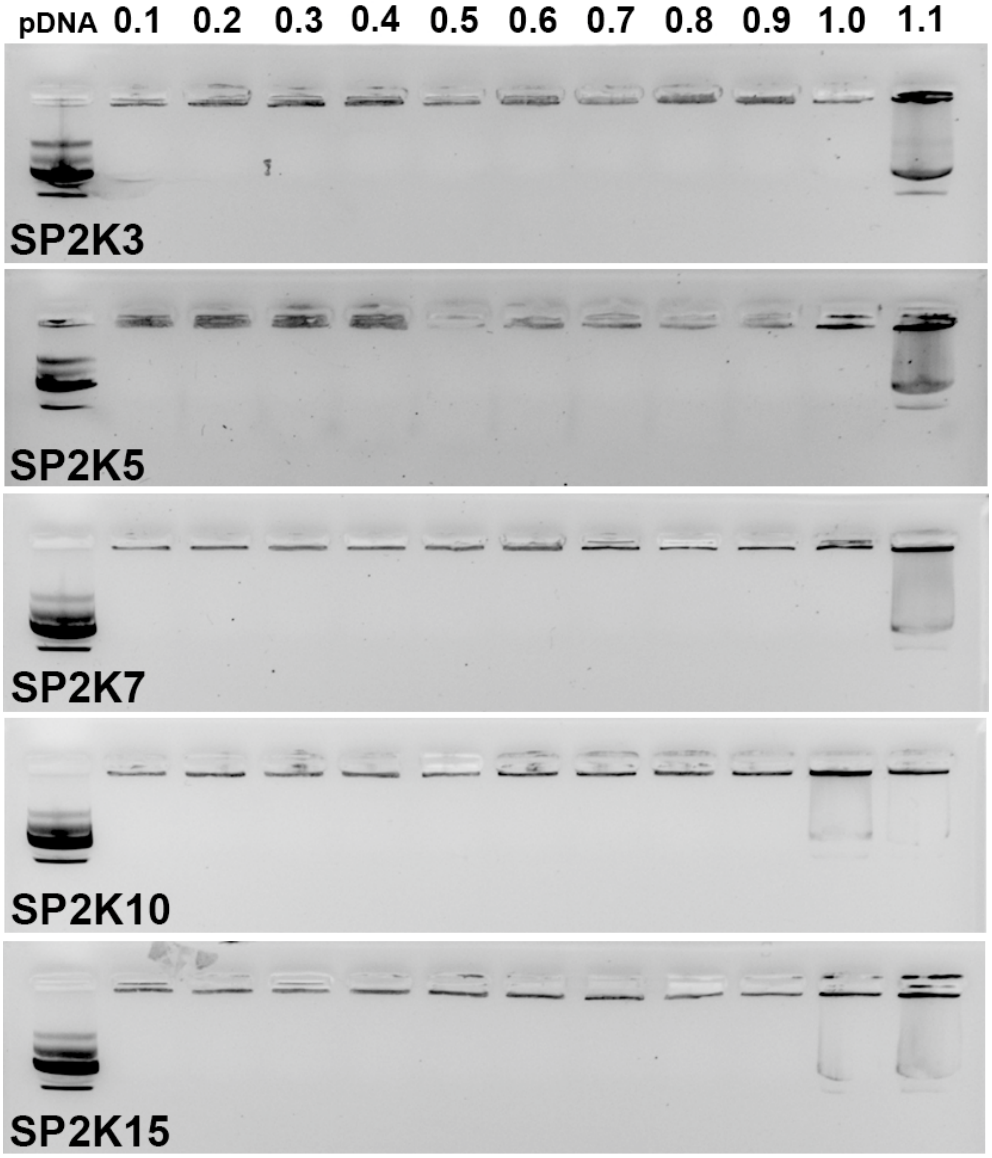

Figure S6. Heparin competition assay of SP2K polyplexes. Numbers mean the concentration (mg/mL) of heparin.

The SP2K polyplexes (0.5 μg pDNA) were prepared in HEPES buffer (pH 7.4) at a weight ratio of 10 after 30 min of incubation at room temperature. Each sample was incubated with heparin solution with various concentration at room temperature. After 30 min of incubation, the agarose gel electrophoresis was run by the identical method to Section 2.5.

A

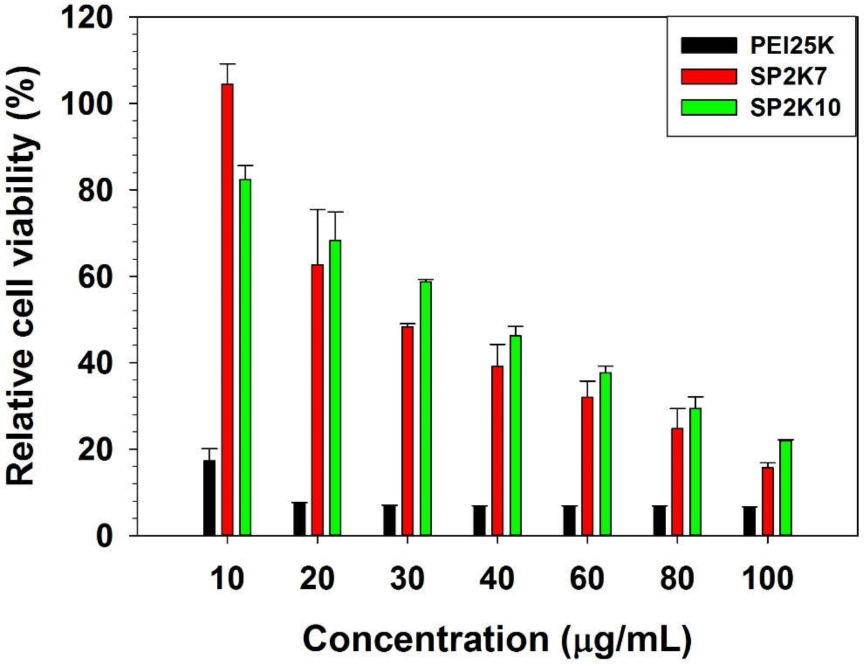

B

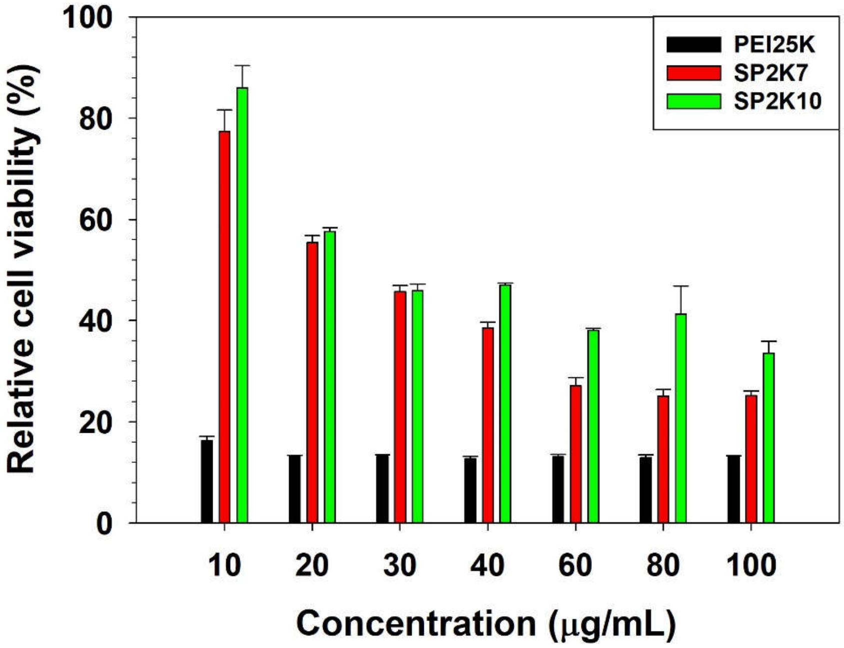

Figure S7. Cytotoxicity of SP2K7 and SP2K10 in (A) A549 cells and (B) RAW264.7 cells.

A549 cells and RAW264.7 cells were maintained in DMEM medium (10% FBS). MTT assay was performed by identical method to Section 2.10. Error bars mean the standard deviations ($n = 3$).

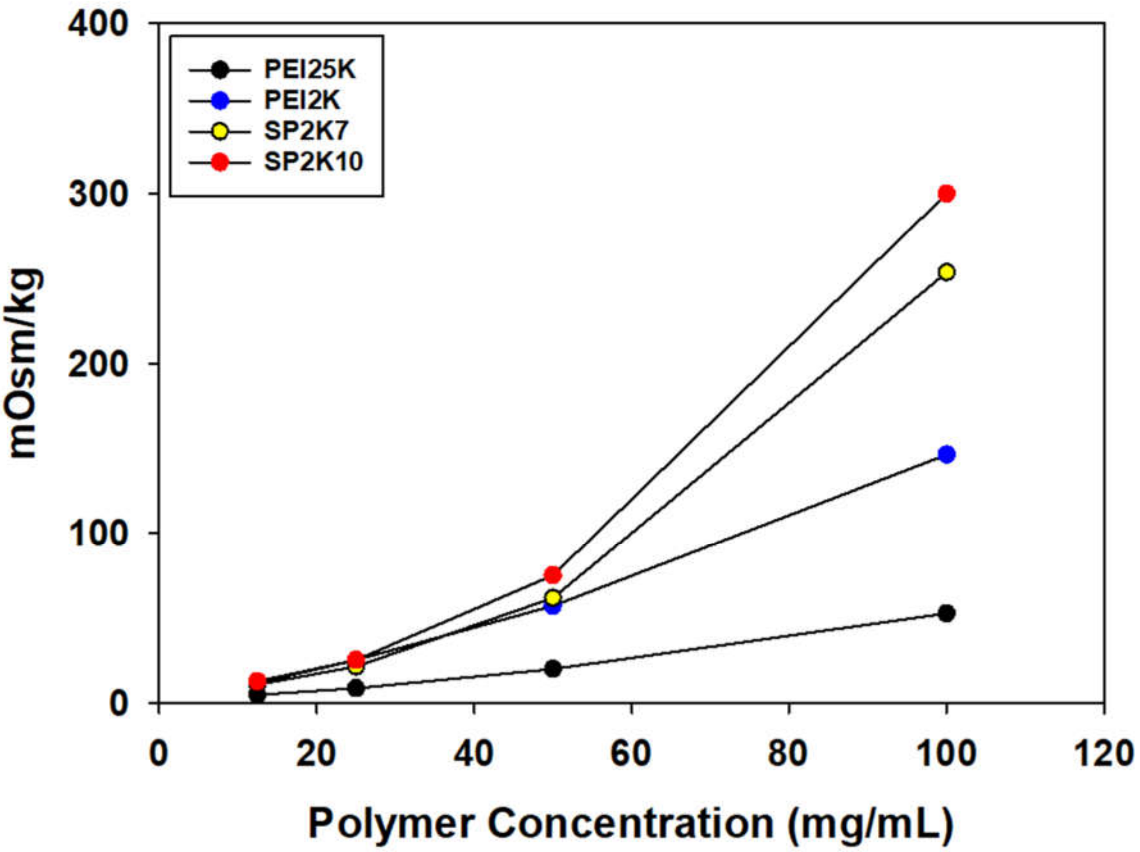

Figure S8. Osmolality of SP2K polymers.

Table 1. Average PDI values of SP2K polyplexes (n=3).

	0.1[a]	0.3	0.5	5	10	20
SP2K3	0.280	0.120	0.347	0.120	0.08	0.129
SP2K5	0.398	0.214	0.212	0.175	0.148	0.132
SP2K7	0.374	0.238	0.189	0.154	0.114	0.121
SP2K10	0.295	0.197	0.152	0.149	0.133	0.127
SP2K15	0.222	0.626	0.12	0.185	0.164	0.117

[a] Numbers mean the weight ratios of SP2K polyplexes.
